# Protocol for a randomized multicenter study for isolated skin vasculitis (ARAMIS) comparing the efficacy of three drugs: azathioprine, colchicine, and dapsone

**DOI:** 10.1186/s13063-020-04285-3

**Published:** 2020-04-28

**Authors:** Robert G. Micheletti, Christian Pagnoux, Roy N. Tamura, Peter C. Grayson, Carol A. McAlear, Renee Borchin, Jeffrey P. Krischer, Peter A. Merkel, for the Vasculitis Clinical Research Consortium

**Affiliations:** 1 Departments of Dermatology and Medicine, University of Pennsylvania, Philadelphia, PA USA; 2 Vasculitis Clinic, Department of Rheumatology, Mount Sinai Hospital, University of Toronto, 60 Murray Street, Toronto, ON M5T3L9 Canada; 3 Health Informatics Institute, University of South Florida, Tampa, FL USA; 4 The National Institute of Arthritis and Musculoskeletal and Skin Diseases, Bethesda, MD USA; 5 Divison of Rheumatology and Department of Biostatistics and Epidemiology, University of Pennsylvania, Philadelphia, PA USA; 6 Department of Biostatistics, University of South Florida, Tampa, FL USA

**Keywords:** Skin vasculitis, Azathioprine, Colchicine, Dapsone, Sequential multiple assignment randomized trial

## Abstract

**Background:**

Skin-limited forms of vasculitis, while lacking systemic manifestations, can persist or recur indefinitely, cause pain, itch, or ulceration, and be complicated by infection or scarring. High-quality evidence on how to treat these conditions is lacking. The aim of this comparative effectiveness study is to determine the optimal management of patients with chronic skin-limited vasculitis.

**Methods:**

ARAMIS is a multicenter, sequential, multiple assignment randomized trial with an enrichment design (SMARTER) aimed at comparing the efficacy of three drugs—azathioprine, colchicine, and dapsone—commonly used to treat various forms of isolated skin vasculitis. ARAMIS will enroll patients with isolated cutaneous small or medium vessel vasculitis, including cutaneous small vessel vasculitis, immunoglobulin A (IgA) vasculitis (skin-limited Henoch-Schönlein purpura), and cutaneous polyarteritis nodosa. Patients not responding to the initial assigned therapy will be re-randomized to one of the remaining two study drugs (Stage 2). Those with intolerance or contraindication to a study drug can be randomized directly into Stage 2. Target enrollment is 90 participants, recruited from international centers affiliated with the Vasculitis Clinical Research Consortium. The number of patients enrolled directly into Stage 2 of the study will be capped at 10% of the total recruitment target. The primary study endpoint is the proportion of participants from the pooled study stages with a response to therapy at month 6, according to the study definition.

**Discussion:**

ARAMIS will help identify effective agents for skin-limited forms of vasculitis, an understudied group of diseases. The SMARTER design may serve as an example for future trials in rare diseases.

**Trial registration:**

ClinicalTrials.gov: NCT02939573. Registered on 18 October 2016.

## Background

Skin-limited subtypes of vasculitis may become chronic or recurrent in up to one third of patients and may persist for several years, with associated discomfort and psychosocial impact [1–4]. Cutaneous vasculitis can be pruritic or painful, may ulcerate and scar or become infected, and may be cosmetically or psychologically disturbing. Despite the chronic and impactful nature of these conditions, high-quality evidence for how best to treat them is lacking. One small randomized controlled trial has been performed to evaluate the efficacy of colchicine [5]. Among 20 patients, after 1 month of follow-up, no significant benefit to colchicine was found compared to placebo. Nonetheless, colchicine is routinely used to treat skin-limited vasculitis, as are azathioprine, dapsone, hydroxychloroquine, methotrexate, and other drugs, based on case series and expert opinion. Absent definitive data, treatment decisions are frequently based on physician preference rather than on patient characteristics or scientific evidence. Dosing and duration of therapies are variable. In lieu of well-defined steroid-sparing options, over-reliance on systemic glucocorticoids may lead to long-term side effects. Topical corticosteroids may relieve itch but fail to prevent new lesions [4, 6–8].

A well-designed comparative effectiveness study of agents frequently used for cutaneous vasculitis could contribute significantly to patient care. The ARAMIS study is a multicenter sequential multiple assignment randomized trial with enrichment (SMARTER) designed to compare the efficacy of three drugs commonly prescribed for skin-limited vasculitis: azathioprine, colchicine, and dapsone. Patients who do not respond to the first assigned study drug, or who have a contraindication or intolerance thereof, may be re-randomized to one of the remaining drugs. This approach increases the power to detect differences between the observed best treatment and the remaining drugs.

## Methods

### Study design

**Fig. 1:**
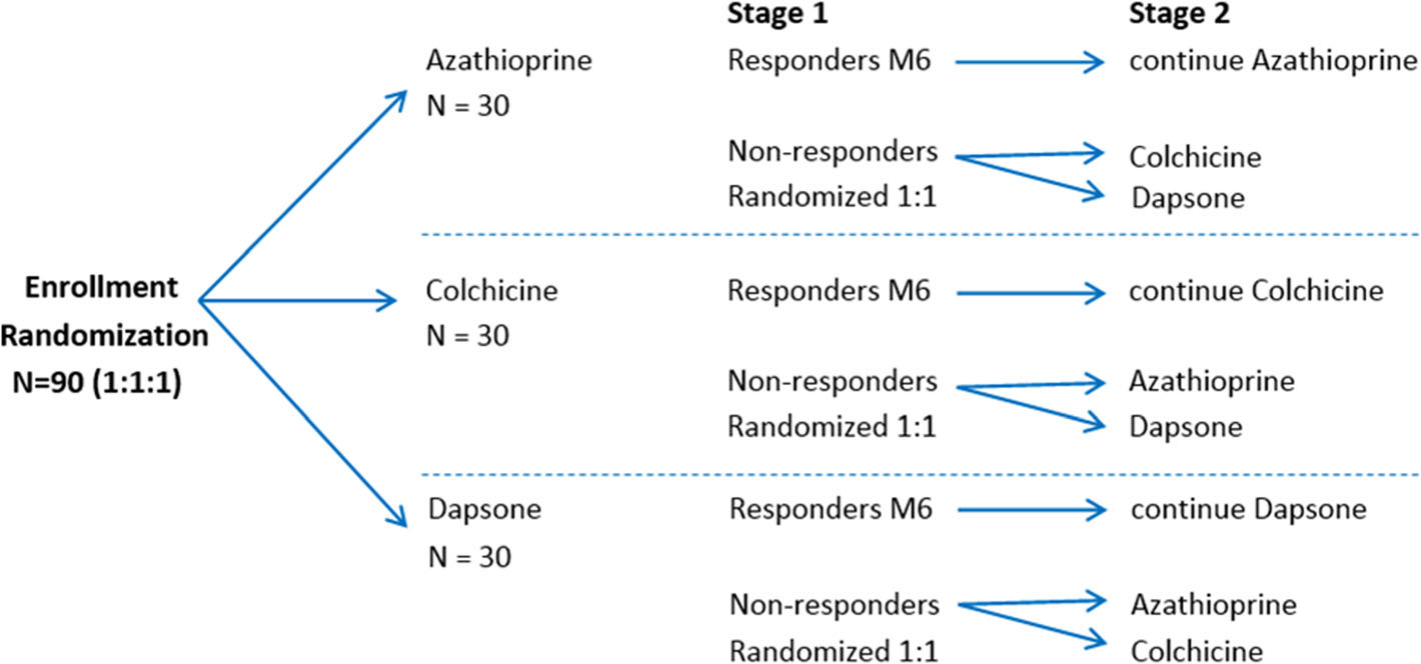
Study design

The study is an open-label, prospective, multicenter SMARTER design comparing the efficacy of azathioprine, colchicine, and dapsone for treatment of isolated skin vasculitis. Patients will be randomized (1:1:1, Stage 1) to receive one of the three study drugs, with response to treatment assessed at month 6 (primary endpoint). Patients who need to discontinue the assigned study drug because of failure, according to the study definition (Table 1), relapse, or an adverse event will be re-randomized (1:1, Stage 2) to receive one of the remaining two study drugs as part of a second 6-month study stage. Patients who have a contraindication to receiving one of the study drugs and those who have been treated previously but did not respond or who had an intolerance to one of the study drugs (per study definition) can be enrolled directly into Stage 2 of the study and randomized to receive one of the two remaining medications. A total of 90 patients are targeted for enrollment. A flow diagram of this trial is shown in Fig. 1.

**Table 1:** Protocol definitions of treatment response and treatment failure

**Response to treatment**:	A complete or significant response to the study drug at month 6. Importantly, the absence of complete or significant response and/or flares of vasculitis occurring between enrollment (or day 1 of Stage 2) and the end of month 3 will not be considered failures; patients must continue taking the assigned study drug until at least the end of month 3, unless they have developed a severe adverse event due to the study drug that warrants its discontinuation. After month 3 (in Stages 1 and 2), the absence of complete or significant response to treatment will be considered a treatment failure *Complete response:* • No new skin lesions of vasculitis within the preceding 3 months *Significant response:* • Three or fewer skin lesions per flare with no more than 1 flare/month and minimally symptomatic, including no significant painful or necrotic lesions, within the preceding 3 months and • Physician global assessment reflecting minimal severity over the prior 28 days (0–2 on a 10-point scale) and • Patient global assessment reflecting an improvement compared to baseline by at least 1 point on a 10-point scale
**Treatment failure:**	A limited response or an absence of response to the study drug at month 6, or the development of a complication of vasculitis at any time after enrollment. The absence of response, a limited response, and/or flares of vasculitis occurring between enrollment (or day 1 of Stage 2) and the end of month 3 will not be considered failures unless the flares require the repeated use of prednisone for ≥ 2 courses *Limited response:* a reduction in the number and/or frequency of new skin lesions of vasculitis and/or in the severity of the lesions but with physician and/or patient global assessment persistently > 2 on a 10-point scale and/or frequency of flares > 1/month *Absence of response:* a complete absence of reduction or an increase in the number and/or frequency of new skin lesions of vasculitis and/or in the severity of the lesions *≥ 2 flares of skin vasculitis requiring prednisone* use between enrollment (or day 1 of Stage 2) and month 3. One course of prednisone for a flare of skin vasculitis for a maximum of 3 weeks is allowed between enrollment or day 1 of Stage 2 and the end of month 3; prednisone is not allowed after month 3 *Complication:* appearance of an extracutaneous manifestation suggesting progression of the underlying vasculitis/condition or an incorrect initial diagnosis of skin-limited vasculitis (at any time between enrollment and month 6). Patients experiencing such complication(s) will have study drug discontinued, undergo a Treatment Stopping Visit, and be treated according to standard of care for their systemic vasculitis

### Participants and recruitment

The participants will be recruited from several international centers affiliated with the Vasculitis Clinical Research Consortium (VCRC), including locations in the USA, Canada, Japan, and other countries.

#### Inclusion criteria

The following are the criteria for inclusion in the trial:

1. Eligible patients will have a diagnosis of isolated skin vasculitis not associated with any significant extracutaneous involvement that would require specific immunosuppressive therapy. Eligible diseases include isolated cutaneous small vessel vasculitis (CSVV), cutaneous polyarteritis nodosa (cPAN), and skin-limited immunoglobulin A (IgA) vasculitis (Henoch-Schönlein purpura) without active or progressing renal involvement, defined as a stable estimated glomerular filtration rate > 60 mL/min, mild and stable or absent microscopic hematuria without red blood cell casts, and mild and stable or absent proteinuria (< 1 g/24 h).
2. The diagnosis must have been confirmed by skin biopsy, including an immunofluorescence study in the case of small vessel vasculitis, at any point prior to enrollment.
3. Participants must have active cutaneous vasculitis lasting for at least 1 month continuously and/or flaring two or more times over the 6 months preceding enrollment. Post-inflammatory lesions such as hyperpigmentation or healing ulceration(s) are not to be considered active vasculitis.
4. Participants must have active/ongoing cutaneous vasculitis lesions at the time of enrollment. Post-inflammatory lesions such as hyperpigmentation or healing ulceration(s) are not to be considered active vasculitis.
5. Participants may have a contraindication to one of the study drugs or may have been treated prior to enrollment with one of the study medications but failed to respond to the medication, according to the study definition of response, or had to stop the medication because of an adverse event. Such patients can be enrolled directly into Stage 2 of the study and be randomized to receive one of the two remaining study drugs.
6. Patients may have received systemic glucocorticoids for their cutaneous vasculitis before enrollment. Patients on a long-term, low, and stable dose of glucocorticoids (≤ 5 mg/day prednisone or equivalent) for other conditions (e.g., asthma) can be enrolled if the likelihood of requiring a dose increase for this other condition is low during the 6-month study period; these patients can remain on a low and stable dose during the study period.
7. Participants must be aged 18 years or older.

Eligibility criteria for Stage 2 (second randomization) of the trial are as follows:

1. Patients entered into Stage 1 of the study who subsequently discontinued the study drug during the 6 months of the first stage of the study or the follow-up period (month 6 to month 12) because of any one of the following endpoints per protocol-defined definitions: a lack of response or failure, a relapse (month 6 to month 12), or a side effect necessitating drug discontinuation
2. Patients with a history of significant intolerance, allergy, or serious adverse events to one of the study medications, deficit in glucose-6-phosphate dehydrogenase (G6PD) or thiopurine methyltransferase (TPMT) or history of hemolytic anemia, or who failed to respond (according to the study definition) to one of the study drugs prior to screening. These patients can be randomized to receive one of the other two study drugs.

#### Exclusion criteria

The following are exclusion criteria for the study:

1. Presence of significant extracutaneous manifestations suggestive of a systemic vasculitis. However, the presence of any of the following findings are *not* exclusion criteria:
  Mild arthralgias, myalgias, peripheral limb edema, fatigue, weight loss ≤ 6 lbs or 3 kg within past 3 months, low-grade fever, mild anemia (hemoglobin ≥10 g/dL)
  For patients with IgA vasculitis: mild and stable microscopic hematuria without red blood cell casts and/or mild and stable proteinuria (< 1 g/24 h) with an estimated glomerular filtration rate > 60 mL/min
2. Known systemic and/or non-skin-isolated vasculitis, such as granulomatosis with polyangiitis (Wegener’s), eosinophilic granulomatosis with polyangiitis (Churg-Strauss), microscopic polyangiitis, systemic polyarteritis nodosa, hypocomplementemic urticarial vasculitis, cryoglobulinemic vasculitis, and patients with detectable anti-neutrophil cytoplasm antibodies (ANCAs) by immunofluorescence or enzyme-linked immunosorbent assay (ELISA)
3. Secondary skin vasculitides such as vasculitis associated with systemic lupus erythematosus, Sjögren syndrome, or other auto-immune condition, cancer, hematological disorder, ongoing active infection, or an ongoing medication. Investigators should consider such underlying diagnoses and perform and interpret appropriate laboratory work-up where indicated based on clinical presentation
4. History of significant intolerance, allergy, or serious adverse events to any of the study medications (such patients can be enrolled directly into Stage 2 of the study and be randomized to receive one of the two other study drugs)
5. Patients who have contraindications to two or more of the study drugs or have been treated prior to enrollment with two or more of the study medications without adequate response or with adverse events requiring drug discontinuation
6. Deficiency in G6PD or history of hemolytic anemia (all patients must be tested for G6PD at the screening visit): such patients can be enrolled directly into Stage 2 and be randomized to receive one of the two other study drugs (azathioprine or colchicine)
7. Known low or absent TPMT activity (testing is not a requirement for study entry). Patients known to have low or absent TPMT can be enrolled directly into Stage 2 and be randomized to receive one of the two other study drugs (colchicine or dapsone)
8. Evidence of significant hepatic insufficiency or liver function tests > 2 times the upper limit of normal
9. Evidence of significant renal insufficiency or estimated glomerular filtration rate < 60 mL/min
10. Evidence of significant or symptomatic anemia or hemoglobin level < 10 g/dL
11. Comorbid condition that has moderate or high likelihood of requiring intermittent courses of prednisone (or equivalent) within the study period, according to the investigator (e.g., chronic obstructive pulmonary disease (COPD), unstable or severe asthma)
12. Current cancer or history of malignancy within the previous 5 years. Patients in remission of a cancer > 5 years or with non-metastatic prostate cancer or treated basal or squamous cell carcinoma of the skin can be enrolled
13. Active uncontrolled or serious infection that may compromise or contraindicate the use of any of the study medications
14. Patient unable to provide informed consent to participate in the study
15. Pregnant or lactating women.

### Interventions

Eligible patients will be randomized by computer to receive one of the three oral medications under investigation for 6 months (primary endpoint). If effective, the medication will be continued, with total follow-up up to month 12. The medications are:

- Azathioprine 2 mg/kg/day (up to 200 mg per day)
- Colchicine 0.6 mg twice per day
- Dapsone 150 mg per day.

Azathioprine will be initiated at 1 mg/kg/day, and if no issues are noted after complete blood count (CBC) testing performed between days 7 and 15, the dose will be increased to 2 mg/kg/day, with subsequent CBC testing 7 to 15 days later, then every 2 weeks for the second and third months of treatment, then monthly or more frequently if dosage alterations occur. Colchicine and dapsone can be started at a lower dose, according to physician and/or patient preference(s), in order to improve initial tolerance to these drugs. However, the target study dose must be achieved within a maximum of 4 weeks after initiation, unless limited by a non-severe adverse event or intolerance to the medication (e.g., diarrhea with colchicine, nausea with azathioprine, or mild anemia on dapsone [drop in hemoglobin level < 2 g/dL with total hemoglobin level still > 10 g/dL]), in which case a lower dose of the study medication may be used. Patients may continue, for the duration of the study, other medications given for non-vasculitis indications.

Patients may have received systemic glucocorticoids for their skin vasculitis before enrollment. For patients on prednisone at the time of enrollment, prednisone should be stopped within a maximum of 6 weeks after initiation of the study drug, as per the predefined tapering schedule (Table 2).

**Table 2:** Schedule of prednisone dose tapering for patients already on prednisone at the time of enrollment. The maximum dose allowed at day 1 of the study is 40 mg/day; the dose of prednisone must be reduced to 40 mg/day prior to enrollment

Time point from enrollment		Starting (study day 1) dose mg/day		
	≥ 30	< 30 to 15	< 15 to 5	< 5 to 1
**Days 2–7**	30	Continue dose	Continue dose	Continue dose
**Days 8–14**	20	15	10 (or continue dose if < 10)	2.5 (or continue dose if < 2.5)
**Days 15–21**	15	10	7.5 (or continue dose if < 7.5)	2.5 (or continue dose if < 2.5)
**Days 22–28**	10	7.5	5	1
**Days 29–34**	7.5	5	2.5	0
**Days 35–40**	5	2.5	1	0
**Days 41–48**	2.5	1	0	0
**Day 49**	0	0	0	0

Patients on long-term, low, and stable doses of glucocorticoids (≤ 5 mg/day prednisone or equivalent) for other conditions (e.g., asthma or adrenal insufficiency) can be enrolled if the likelihood of requiring a dose increase for the other condition is low and minimal during the 6-month study period (these patients will remain on the low and stable dose during the study period, with the option to receive one short course of prednisone at higher doses for skin vasculitis flare during the first 10 weeks of the study period

From the beginning of Stage 1 or Stage 2 until week 10 of the stage, patients who experience a flare of skin vasculitis can be given one short course of prednisone for a maximum of 19 days, according to one of two predefined regimens (Table 3), chosen based on physician preference and patient and disease characteristics.

**Table 3:** Protocol glucocorticoid dose-tapering regimens for patients given prednisone during the first 10 weeks (of each study stage) for a skin vasculitis flare

Time from initiation	Daily dose Option 1	Daily dose Option 2
Days 1–3	30 mg/day	10 mg/day
Days 4–6	20 mg/day	7.5 mg/day
Days 7–9	15 mg/day	5 mg/day
Days 10–12	10 mg/day	2.5 mg/day
Days 13–15	5 mg/day	0
Days 16–18	2.5 mg/day	0
Day 19	0	0

At week 12 (Stages 1 and 2), all patients must be off prednisone

For patients requiring a second course of prednisone for vasculitis flare, the study drug will be considered a treatment failure

### Outcomes

#### Primary outcome

The primary study endpoint will be the proportion of participants with a response to therapy (according to the study definitions outlined in Table 1) at month 6 of the pooled study Stages 1 and 2.

#### Secondary outcomes

The secondary study endpoints include:

1. Proportion of patients with complete response to therapy (no new lesions within the preceding 3 months) at months 3, 6, and 12
2. Proportion of patients with significant response to therapy (three or fewer minimally symptomatic lesions per flare, with no more than one flare per month in the preceding 3 months) at months 3, 6, and 12
3. Time to achieve complete or significant response
4. Time to vasculitis flare for patients who achieved a complete or significant response before month 6 but subsequently relapsed
5. Frequency of vasculitis flares/new lesions compared to baseline
6. Physicians’ global assessments of response
7. Patients’ global assessments of response
8. Prednisone use during the study period
9. Skindex29 score at months 1, 3, 6, 9, and 12 (captures skin-related pain, pruritus, discomfort) [9, 10]
10. Patient-reported outcomes/disease burden (36-item Short Form survey [SF-36], Patient-Reported Outcomes Measurement Information System [PROMIS], Health-Related Quality of Life assessment) at months 1, 3, 6, 9, and 12
11. Response according to patient and disease characteristics
12. Grading of standardized photographs by blinded investigators.

### Participant timeline and monitoring

Participants will have a minimum of six study visits at the study site, including a screening visit, a baseline visit (which can be combined with the screening visit and randomization on the same day), and then visits at months 1, 3, 6, 9, and 12 if they respond to the Stage 1 study drug.

If subsequently entered into Stage 2, patients will be seen at the study site following a schedule similar to that for Stage 1: day 1 of Stage 2 (baseline/randomization), then at months 1, 3, 6, 9, and 12. If entered directly in Stage 2, patients will be seen at the study site for a screening visit, a baseline visit (which can be combined with the screening visit and randomization), and then visits at months 1, 3, 6, 9, and 12.

Prior to enrollment, all patients will be screened for G6PD deficiency. TPMT testing is not routine standard of care at all centers; patients can be tested for TPMT activity before enrollment or not, according to local practice. Patients known to have low or absent TPMT can be enrolled directly into Stage 2 of the study and be randomized to receive one of the two other study drugs (colchicine or dapsone). All patients on azathioprine will start at 1 mg/kg/day and will have close CBC monitoring, as detailed above, regardless of their TPMT testing [11, 12].

Laboratory monitoring during the study will include, for azathioprine and dapsone, a minimum of CBC monitoring every 2 weeks for the first 3 months of therapy, then monthly or more frequently if dosage alterations occur. If there is any significant change in key laboratory parameters from baseline (including a reduction in estimated glomerular filtration rate to < 60 mL/min, transaminase level > 2 times the upper limit of normal, hemoglobin reduction > 2 g/dL, or hemoglobin < 10 g/dL), the test should be repeated, and, if the change is confirmed, the medication will be discontinued with reporting of an adverse event. Patients are required to keep drug diaries throughout the study to document adherence to the course of therapy.

Additional study visits will occur in the case of a disease flare or an adverse event. The schedule for data collection within the study is outlined in Table 4.

**Table 4:** Data collection schedule for on-site visits (for the two study stages)

Data forms	Screening visit	Randomization^a^ Day 1 Stage 1/Day 1 of Stage 2 (direct entry)^a^	For Stage 1 failures with subsequent entry in Stage 2	Month 1 visit	Month 3 visit	Month 6 visit^g^	Month 9 visit	Month 12 visit	Treatment Stopping Visit^h^
Informed consent	X								
Eligibility review	X								
Demographics	X								
Baseline medical history	X								
Labs: CBC,^b^ chemistry panel, liver function tests, urinalysis, and other relevant laboratory tests^c^	X		X	X	X	X	X	X	X
G6PD screening test	X								
Urine pregnancy test^d^	X								
Follow-up medical history			X	X	X	X	X	X	X
Baseline vasculitis medication form	X								
Non-vasculitis medication form	X		X	X	X	X	X	X	X
Follow-up vasculitis medication form			X	X	X	X	X	X	X
Baseline comorbidity form	X								
Follow-up comorbidity form						X		X	X
Drug diary and prednisone form		X	X	X	X	X	X		
Patient global assessment	X		X	X	X	X	X	X	X
Physician global assessment	X		X	X	X	X	X	X	X
PROMIS	X		X	X	X	X	X	X	X
SF-36	X		X	X	X	X	X	X	X
Skindex	X		X	X	X	X	X	X	X
Standardized photographs^e^	X		X	X	X	X	X	X	X
As necessary^f^									
Adverse event report		X	X	X	X	X	X	X	X
Diagnostic sinus & chest imaging form	X								
Protocol deviation									
Hospitalization									
Death record									
Primary outcome of study						X			
Study therapy discontinuation									
Protocol termination form									

Stage 1 lasts up to 12 months. Subjects who fail Stage 1 will be re-randomized and enter Stage 2. Stage 2 lasts up to 12 months and restarts the visit schedule to month 1, month 3, etc., as outlined above

^a^Randomization can occur remotely, see section 4div. of the protocol for details. Visits for months 1, 3, 6, 9, 12 have a +/− 2-week window

^b^CBC monitoring between days 7 and 15 after starting azathioprine, then again 7 to 15 days later, continue monitoring every 2 weeks for months 2 and 3 of treatment, then monthly or more frequently if dosage alterations or other therapy changes; CBC monitoring at minimum of every 2 weeks for first 3 months, per standard practice for those receiving dapsone; CBC monitoring at minimum of every 3 months for those receiving colchicine. CBC monitoring applies to both Stage 1 and Stage 2

^c^Results of labs drawn within 1 month prior to screening can be utilized to determine eligibility

^d^Urine pregnancy test is only for women of child-bearing potential

^e^See protocol section 4fi for additional information on photographs

^f^In case of a severe adverse event, protocol deviation, or event corresponding to a study failure, efforts will be made to see the patient in person at the investigator study site as soon as possible

^g^Primary study endpoint is at month 6 of each stage, but follow-up is planned until month 12 (secondary outcomes)

^h^The Treatment Stopping Visit should only be completed for subjects meeting the criteria listed in section 4h of the protocol

If the patient experiences an endpoint defined as failure of treatment in Stage 2, he/she will be treated according to his/her physician’s discretion, outside of the study.

### Randomization method and stratification

Patients eligible for enrollment in Stage 1 will be randomized (1:1:1) to receive one of the three medications under investigation for 6 months. The endpoint is response to treatment at month 6 (Stage 1). If the patient is entered into Stage 2 (directly or because he/she discontinued the initial study drug because of a lack of response, treatment failure, or drug side effect), he/she will be randomized to receive one of the two remaining study drugs (1:1). The endpoint in Stage 2 is the response to treatment at month 6.

Randomization will be stratified at each stage according to:

- IgA vasculitis versus other forms of vasculitis
- Use of prednisone prior to and at the time of randomization versus no pre-enrollment use of prednisone.

### Sample size

The statistical rationale and construct of the proposed design have been detailed elsewhere [13]. The aim of this design is, by pooling the results of the two study stages, to increase the power of the study to detect a difference between the arms. The primary hypothesis is that one of the study drugs will achieve a response in 50% of the patients at month 6 (Stages 1 and 2 combined) compared to only 25% of those receiving the best of the remaining two drugs. Under these assumptions, with an initial plan to enroll 90 participants (30 in each arm of the first study stage), the pooled analysis of the two study stages (first randomization and, for non-respondents, second randomization) will have 68% power to detect a significant difference between the study drugs (two-sided alpha = 0.10). The number of patients enrolled directly in Stage 2 of the study will be capped at 10 (10% of the total recruitment target for the study).

Because skin vasculitis is a rare disease, an interim analysis will be conducted after enrollment of 45 patients (50% of the planned accrual target) in order to determine, based on the response rate in each arm, whether an adjustment in the study design and/or enrollment target is possible and/or needed. Options might include early closure of the study for futility or deletion of one study arm for excessive rate of treatment failure (drop-the-loser design), with or without an accompanying increase in the sample size of the remaining arms.

### Data management

Clinical information will be collected and stored for this study according to the study data collection schedule, Table 4. This information is collected as part of routine clinical practice and, therefore, is not specifically mandated by the protocol:

- Medical history data
- Physical examination data, including detailed descriptions of skin lesions
- CBC, tests of renal and liver function, urinalysis, and other laboratory tests relevant to disease assessment or adverse event monitoring
- Chest radiographs and other diagnostic images.

All study data will be collected via systems and online electronic case report forms created in collaboration with the Rare Diseases Clinical Research Network Data Management and Coordinating Center and will comply with all applicable guidelines regarding patient confidentiality and data integrity using encrypted communication links. Online collection forms and procedures have been designed to ensure optimal data quality for this study and incorporate reasonable checks to minimize transcription and omission errors.

### Statistical analysis

Descriptive analyses of the participants and their skin vasculitis before and at the time of enrollment will be conducted.

The primary and secondary outcome endpoints will be estimated for each drug and each of the two stages of the study. The primary comparison will be the comparison of the best treatment arm in terms of Stage 1 response versus the maximum of the remaining two treatment arms. The inference for this comparison will be based on a weighted average of the Stage 1 and Stage 2 differences in response rates. An a priori weight of 0.70 to Stage 1 results has yielded the maximum power in simulations and will be used in the analysis [13].

Other continuous efficacy outcomes will also be estimated for each treatment arm in each stage. The inference for any pairwise comparison will be from a general linear model with independent factors of treatment and stage and the change from baseline as the dependent variable.

A descriptive analysis of the participants withdrawn from the study for treatment failure or any other reason will also be conducted. All patients will have follow-up visits at month 12.

### Ethical considerations

The proposed research study is a randomized clinical trial to evaluate three different drugs that are all commonly used to treat skin vasculitis. The risks of participating in this study are expected to be low and will be due to possible side effects of the study drugs, which are all routinely used to treat the condition under study (skin-limited vasculitis).

Written informed consent will be obtained from all participants by the principal investigator. Participants are free to withdraw from the study at any time without compromising their relationship with their physician or their future medical care.

Registration of participants in this study will employ an interactive online database. The Data Management and Coordinating Center will use a system of coded identifiers to protect participant confidentiality and safety. Only the registering site will have access to the linkage between the local number and the personal participant ID number of the subject.

Study participants are instructed to contact their doctor immediately to report clinical deterioration between regular health checks, or at any time they want a consultation to discuss side effects or other health-related questions. Rapid access to specialist care is guaranteed for all study groups.

The trial was approved by the Vasculitis Clinical Research Consortium (VCRC) Data and Safety Monitoring Board of the National Institute of Arthritis and Musculoskeletal and Skin Diseases. The trial was registered on 18 October 2016 in the ClinicalTrials.gov database (https://clinicaltrials.gov) with identification number NCT02939573. The investigators plan to publish results of this trial, when complete, in a peer-reviewed journal.

## Discussion

If successful, the ARAMIS trial will help identify effective agent(s) for treatment of primary cutaneous vasculitis, providing a major therapeutic advancement in an area of unmet need. ARAMIS may also serve as an example of feasible SMART designs for future trials of rare diseases other than skin vasculitis. The original and innovative study design, successfully employed in oncology, has not previously been utilized for the study of vasculitis or connective tissue diseases.

Chronic or recurrent isolated skin vasculitis is rare. Inclusion/exclusion criteria have been designed to mimic real-world scenarios and be minimally restrictive. ARAMIS is a multicenter, international study to be conducted through the VCRC with support from the Canadian Vasculitis research network (CanVasc).

ARAMIS relies on a SMART design adapted to a smaller patient sample size, with enrichment (SMARTER) [13]. SMARTs are most often used with large patient populations and well-established therapeutic options to determine the optimal sequences of therapies. The objectives are to determine the best treatment in the initial stage and, by pooling results of the other study stages, make inferences about the treatments in comparison to one another. Re-randomization of non-responders in the ARAMIS study will increase the power to detect differences between drugs. Despite the use of an adapted SMARTER design, the pooled analysis of the two ARAMIS study stages will only have 68% power to detect a significant difference between the study drugs. Whereas this power might be sufficient to detect a major difference, ARAMIS is a pilot study which explores a novel design and may serve as a preliminary trial before a possible larger one.

ARAMIS will be crucial to the development of a global research program on skin vasculitis, aimed at deciphering its pathogenesis and determining its optimal treatment, based on broad partnership between dermatologists, rheumatologists, and other specialists with an interest in vasculitis.

### Trial status

As of November 2019, ARAMIS has received ethical approval and is enrolling patients at 10 sites in the United States and four in Canada. The most recent version of the protocol (dated 11 March 2019) was approved by the University of Pennsylvania Institutional Review Board on 10 June 2019. Recruitment began on 7 August 2017. The trial is expected to be completed by the end of December 2020.

## Data Availability

Data will be made publicly available at the conclusion of the trial and can be obtained from the corresponding author on request.

## Abbreviations

G6PD: Glucose-6-phosphate dehydrogenase
IgA: Immunoglobulin A
SMART: Sequential multiple assignment randomized trial
TPMT: Thiopurine methyltransferase
VCRC: Vasculitis Clinical Research Consortium

## References

[1] Callen JP (1998). Cutaneous vasculitis: what have we learned in the past 20 years?. Arch Dermatol.

[2] Callen JP (1999). A clinical approach to the vasculitis patient in the dermatologic office. Clin Dermatol.

[3] Carlson JA, Chen KR (2007). Cutaneous vasculitis update: neutrophilic muscular vessel and eosinophilic, granulomatous, and lymphocytic vasculitis syndromes. Am J Dermatopathol.

[4] Chen KR, Carlson JA (2008). Clinical approach to cutaneous vasculitis. Am J Clin Dermatol.

[5] Sais G, Vidaller A, Jucgla A, Gallardo F, Peyri J (1995). Colchicine in the treatment of cutaneous leukocytoclastic vasculitis. Results of a prospective, randomized controlled trial. Arch Dermatol.

[6] Gibson LE (2001). Cutaneous vasculitis update. Dermatol Clin.

[7] Gonzalez-Gay MA, Garcia-Porrua C, Pujol RM (2005). Clinical approach to cutaneous vasculitis. Curr Opin Rheumatol.

[8] Pina T, Blanco R, Gonzalez-Gay MA (2013). Cutaneous vasculitis: a rheumatologist perspective. Curr Allergy Asthma Rep.

[9] Chren MM (2012). The Skindex instruments to measure the effects of skin disease on quality of life. Dermatol Clin.

[10] Chren MM, Lasek RJ, Quinn LM, Mostow EN, Zyzanski SJ (1996). Skindex, a quality-of-life measure for patients with skin disease: reliability, validity, and responsiveness. J Invest Dermatol.

[11] Stassen PM, Derks RP, Kallenberg CG, Stegeman CA (2009). Thiopurinemethyltransferase (TPMT) genotype and TPMT activity in patients with anti-neutrophil cytoplasmic antibody-associated vasculitis: relation to azathioprine maintenance treatment and adverse effects. Ann Rheum Dis.

[12] Jabin D, Kumar S, Gow PJ (2010). Outcome of patients on azathioprine: a need for a better pre-treatment assessment and dosing guideline. N Z Med J.

[13] Tamura RN, Krischer JP, Pagnoux C, Micheletti R, Grayson PC, Chen YF (2016). A small n sequential multiple assignment randomized trial design for use in rare disease research. Contemp Clin Trials.

